# Synthesis, Structural, and Biological Studies of Some Schiff Bases and Their Metal Complexes

**DOI:** 10.1155/2008/875410

**Published:** 2008-07-27

**Authors:** A. P. Mishra, Monika Soni

**Affiliations:** Department of Chemistry, Dr. Harisingh Gour University, Sagar (M.P.) 470003, India

## Abstract

New bidentate or tridentate Schiff bases and their VO(II) and Co(II) complexes formed by the condensation of methyl isobutyl ketone with nicotinamide (mna)/2-amino-4-chlorophenol (map) and 2-hydroxy acetophenone with nicotinamide (han)/isoniazide (hai). Physicochemical characterization has been carried out to determine the structure of the complexes. The FAB mass and thermal data show degradation pattern of the complexes. XRD analysis reveals that all the studied complexes crystallize as tetragonal crystal system. Some of the complexes have been screened for their antimicrobial activity by the well diffusion technique using DMSO as solvent on different species of pathogenic bacteria/fungi, that is, *E. coli, S. aureus, S. fecalis, A. niger, T. polysporum*, and their antimicrobial potency have been discussed. It has been found that all the complexes are antimicrobially active and show higher activity than the free ligand. Metal chelation affects significantly the antimicrobial/bioactive behavior of the organic ligands.

## 1. INTRODUCTION

Research interest in V/O chemistry derives from its utility in several biological and industrial processes [1]. The coordination chemistry of vanadium has acquired renewed interest since the discovery of vanadium in organisms such as certain ascidians and Amanita mushrooms and as a constituent of the cofactors in vanadate-dependent haloperoxidases and vanadium nitroginase [2]. Recent advances in catalytic and medicinal properties of vanadium complexes have stimulated their design and synthesis. The biochemical aspects of vanadium complexes have further promoted the coordination chemistry of vanadium [3]. Its biological significance is further examplified by its incorporation in natural products and enzyme in potent inhibitor of phosphoryl transfer. Vanadium-containing compounds have their utility as insulin mimetic and antiamoebic agent. The potential of vanadium (V) complexes as antiamoebic agents has thus far only been marginally explored [4]. It is also suggested that vanadium could be considered as a representative of a new class of nonplatinum metal antitumor agents.

Schiff bases and their complexes have a variety of applications in biological clinical and analytical fields [5]. Recently there has been a considerable interest in the chemistry of hydrazine and hydrazone compounds because of their potential pharmacological applications [6]. The remarkable biological activity of acid hydrazides R–CO–NH–NH_2_, their corresponding aryolhydrazones R–CO–NH–N=CHR, and also their mode of chelation with transition metal ions has aroused interest in the past due to possible biomimetic applications. The coordination compounds of aroylhydrazones have been reported to act as enzyme inhibitors and are useful due to their pharmacological applications [7]. In the present paper, we describe the synthesis, characterization, and biological activity of some oxovanadium (IV) and cobalt (II) complexes of Schiff bases, namely, *mna, map, han, and hai*.

## 2. EXPERIMENTAL

### 2.1. Synthesis of Schiff bases (ligands) and complexes

Schiff bases (mna, han, hai, map) have been synthesized by condensing the methanolic solution of methyl isobutyl ketone (0.08 mol) to the methanolic solution of nicotinamide/2-amino-4-chlorophenol (0.08 mol) and the methanolic solution of 2-hydroxy acetophenon (0.08 mol) with the methanolic solution of nicotinamide/isoniazid (0.08 mol) in equimolar ratio. The condensation product was filtered, washed with ethanol and ether, recrystalised with ethanol, and dried under reduced pressure over anhydrous CaCl_2_. Purity of the compounds was monitored by TLC using silica gel G. Schiff bases have been characterized by elemental and IR spectra.

The VO(II) and Co(II) complexes have been prepared by mixing the methanolic solution of VOSO_4_ · 5H_2_O/CoCl_2_ · 6H_2_O (0.08 mol) to the methanolic solution of Schiff bases (mna, han, hai, map) (0.016 mol) in 1 : 2 molar ratio. The resulting mixture was then refluxed on water bath for 10–12 hours. The precipitated complexes were recrystallized twice with ethanol, finally washed with petroleum ether (60–80°C), and dried under reduced pressure over anhydrous CaCl_2_ in a dessicator.

### 2.2. Characterization of the complexes

The microanalyses % C, N, and H are estimated (on Heraeus elemental analyzer), and IR spectra were recorded (on Perkin Elmer RX-I Spectrophotometer) from Lucknow. Room temperature molar conductance (on Elico-CM82 Conductivity Bridge) and electronic absorption measurements (on Perkin Elmer Lambda-2B spectrophotometer) have been done from Sagar. TGA (on mettler Toledo star e system) has been done from Chandigarh, X-ray from Nagpur, FAB mass (on JEOL SX102/DA-6000 mass spectrometer/data system using argon/xenon (accelerating voltage 10 kV) from Lucknow. X-band EPR spectra were recorded at room temperature on Varian E-112 spectrophotometer (TCNA (*g* = 2.0027) as the standard) from Mumbai.

## 3. RESULTS AND DISCUSSION

The analytical and physical data of the metal complexes are presented in Table 1. Elemental analysis of the complexes indicates the stoichiometry to be 1:2 metal: ligand (Schiff base). The molar conductance values in methanol (10^−3^ M) are 124.5 and 53.8 S cm^2^ mol^−1^, respectively, for Co(II) and VO(II) (mna) complexes which indicate the uni-bivalent electrolytic nature of the complexes. The observed conductance values for Co(II) and VO(II) (han), (hai), and (map) complexes fall in the range of 6.5–20.1 S cm^2^ mol^−1^ suggesting the nonelectrolytic nature of the complexes.

## 4. THERMAL ANALYSIS

### 4.1. Thermal decomposition of [VO(mna)_**2**_] SO_**4**_ · 2H_**2**_O [2]

The TG curve of the complex shows that the complex starts decomposing at 60°C. Elimination of lattice water molecules has been observed on increasing the temperature up to 130°C (Re. wt%, obs./cal., 95/93.7). Above this temperature a gradual (but slow) weight loss continues up to 450°C, which corresponds to the decomposition of the Schiff base and sulphate moiety from the metal chelate [12]. Almost horizontal thermal curve has been observed after 450°C. The remaining weight (obs./cal. 29/24.6) corresponds to a mixture of metal oxide in nitrogen atmosphere and some ashes as ultimate pyrolysis product.

## 5. FAB MASS SPECTRA

The FAB mass spectrum of [Co(han)_2_] [6] shows a molecular ion peak (M^+^) at m/z 545 suggesting the complex to be monomeric. The spectrum of complex also shows a series of peaks at m/z 513, 460, 391, 338, 276, 107 corresponding to various fragments [2, 13]. Their intensity gives an idea about the abundance and stability of the fragments. On the basis of the above spectral studies, the following molecular formula (see Table 1 [6]) may be suggested for this complex.

The FAB mass spectrum of [VO(map)_2_] · 5H_2_O [14] shows a molecular ion peak (M^+^) at m/z 579, which suggests the monomeric nature of the complex and confirms the proposed formula [14]. The peaks of appreciable intensity have been observed at m/z values 560, 519, 503, 487, 276, and 107, which indicate the fragmentation pattern. The m/z value 560 corresponds to [VO(map)_2_] · 4H_2_O, 519 to [VO(map)_2_]2H_2_O, 503 to [VO(map)_2_] · H_2_O, 487 to [VO(map)_2_], 276 to [VO(map)]. The value 107 corresponds to VO with chelated O and N donor as ligand moiety [2, 11, 13].

## 6. INFRARED SPECTRA

### 6.1. Complexes of Oxovanadium (IV) and Cobalt (II) with Methyl isobutyl ketone nicotinamide (mna)

IR spectrum shows band at 1684 cm^−1^ *ν*(C=O); this has shifted to lower frequency region (1676 ± 10 cm^−1^) in the complexes indicating the participation of amide (C=O) group in chelation. The ligand band at 1620 cm^−1^ due to *ν*(C=N) azomethine group has shifted to lower frequency (1590 ± 2 cm^−1^) in the complexes indicating coordination through azomethine nitrogen. The appearance of broad bands at 3350 and 3380 cm^−1^ in the spectra of complexes has been assigned to associate water molecules [14, 15]. A medium intensity band at 655 cm^−1^ in Co(II) complex is assignable to rocking mode due to coordinated water molecule. Some new bands of weaker intensity at 520 ± 6 cm^−1^ and 469 ± 10 cm^−1^, in both the complexes, give inferences about *ν*(M–O) and *ν*(M–N) bonding. The characteristic band at 972 cm^−1^ in VO(II) complex has been assigned to *ν*(V=O) vibrations [3]. The presence of an ionic sulphate group in VO(II) complex has been confirmed by the appearance of the three bands [16, 17] at 1119(*ν*_3_) cm^−1^, 900(*ν*_1_), and 618(*ν*_4_)cm^−1^.

### 6.2. Complexes of Oxovanadium (IV) and Cobalt (II) with 2-Hydroxy acetopenone-nicotinamide (han)

Schiff base exhibits a strong intensity band at 1683 cm^−1^ due to C=O (amide) and this has shifted to lower side (1675 cm^−1^) in Co(II) complex, suggesting the chelation through carbonyl-oxygen atom of the free base. A medium intensity band in ligand spectra at 1618 cm^−1^ is attributed to *ν*(C=N) azomethine mode. In both complexes, this band has shifted to higher frequency (1635 ± 6 cm^−1^), suggesting its involvement in chelation [18]. Another important ligand band, occurring at about 1350 cm^−1^ due to phenolic-OH, has been found absent in complexes. This indicates the deprotonation of phenolic-OH on coordination with metal. A band at 1202 cm^−1^ due to phenolic C–O shifts to higher side (1220 ± 10 cm^−1^) in the complexes. This substantiates the same view. The appearance of broad band around 3410 cm^−1^ in the spectra of VO(II) complex has been assigned to associated water molecule. The new weak bands at 420 ± 10 and 510 ± 2 cm^−1^ are due to the formation of *ν*(M–N) and *ν*(M–O) bands. In VO(II) complex, a characteristic nonligand sharp band at 972 cm^−1^ is due to V=O vibrations [10, 11, 16, 17].

### 6.3. Complexes of Oxovanadium (IV) and Cobalt (II) with 2-Hydroxy acetophenone-isoniazide (hai)

IR spectrum exhibits a strong band at 1682 cm^−1^ due to (C=O) amide group. This has shifted down (1653 cm^−1^) in the spectra of Co(II) complex indicating coordination through the carbonyl oxygen. A band at 1607 cm^−1^ due to *ν*(C=N) azomethine group has shifted down at 1593 ± 10 cm^−1^ in both complexes. This suggests the involvement of the azomethine group in coordination. A band at 1373 cm^−1^ due to phenolic-OH deformation has been found absent in complexes. This indicates the deprotonation of phenolic-OH on coordination with metal ions [19–21]. A strong band at 1281 cm^−1^ in ligand spectrum due to C–O shifts to higher side (1325 ± 3 cm^−1^) in complexes. This substantiates the same view [20]. The appearance of broad band around 3310 ± 45 cm^−1^ in the spectra of complexes has been assigned to associated water molecules. The new weaker bands at 533 ± 8 and 420 ± 18 cm^−1^ in the metal complexes have been assigned to *ν*(M–O) and *ν*(M–N) modes, respectively. A very sharp peak at 970 cm^−1^ suggests the presence of V=O bond in VO(II) complex [10, 11, 16, 17].

### 6.4. Complexes of Oxovanadium (IV) and Cobalt (II) with Methyl isobutyl ketone-2-amino-4-chloro phenol (map)

The ligand spectrum exhibits bands at 3380 cm^−1^ and 1386 cm^−1^ due to phenolic-OH. These bands are absent in the spectra of the complexes, indicating the deprotonation of phenolic-OH on coordination with metal ions. An intense ligand band at 1278 cm^−1^ (phenolic-C–O) has shifted to higher frequency side by 10–20 cm^−1^, in metal complexes. This further supports the coordination of phenolic oxygen with metal ions. The ligand band at 1604 cm^−1^ (due to C=N) has shifted to lower frequency (1560 ± 6 cm^−1^) in the complexes, indicating coordination through azomethine nitrogen [22, 23]. The appearance of broad band around 3186 ± 24 cm^−1^ in the spectra of complexes has been assigned to associated water molecules. A medium intensity band at 745 cm^−1^ in the spectrum of Co(II) complex is assignable to rocking mode due to coordinated water molecule. Some new bands of weaker intensity in complexes at 540 ± 5 cm^−1^ and 430 ± 5 cm^−1^ give inference about *ν*(M–O) and *ν*(M–N) bonding. A nonligand sharp band at 983 cm^−1^ in the spectrum of VO(II) complex is assignable to *ν*V=O [10, 11, 16, 17].

## 7. ESR SPECTRA OF THE OXOVANADIUM (IV) COMPLEXES 

The X-band EPR spectra of oxovanadium (IV) (*d^1^*, ^51^V, I = 7/2) complexes are not so resolved at room temperature to exhibit all the eight-hyperfine lines. The calculated values of *g*_||_, *g*_⊥_, *g*_av_, and Δ*g* for these two complexes are given in Table 3. Here, *g*_av_ = 1/3[2*g*_⊥_ + *g*_||_]. The values are typical of the spectra displayed by trigonal bipyramidal or square pyramidal VO(II) complexes with one unpaired electron in an orbital of mostly *d_xy_* character. An anisotropic EPR spectrum is expected to exhibit two *g* values (*g*_*z*_ = *g*_||_ < *g*_⊥_ = *g*_*x*_ = *g*_*y*_) [2, 11, 20, 24, 25].

## 8. X-RAY STUDIES

X-ray powder diffractogram of [Co(hai)_2_] · 2H_2_O [13] and [VO(map)_2_] · 5H_2_O [14] has been recorded using CuK*α* as source in the range 5.50–80° (2*θ*). X-ray crystal system has been worked out by trial and error methods, for finding the best fit between observed and calculated sin^2^ *θ* values [2]. Crystal parameters for [Co(hai)_2_] · 2H_2_O [13] complex are as *a* = *b* = 17.2238 Å, *c* = 30.4478 Å, V = 9032.62 Å [3], Z = 9, Dobs = 1.000 g/cm^3^, Dcal = 1.030 g/cm^3^, particle size = 21.87 nm, and crystal parameters for [VO(map)_2_] · 5H_2_O [14] complex are as *a* = *b* = 11.2836 Å, *c* = 34.4447 Å, V = 4385.86 Å [3], Z = 7, Dobs = 1.5367 g/cm^3^, Dcal = 1.6951 g/cm^3^, particle size = 12.01 nm. This reflects that these complexes have crystallized in tetragonal system.

## 9. BIOLOGICAL ACTIVITY

The in vitro biological [26] screening results are given in Tables 4 and 5.

These observations show that the majority of the compounds are more active than their respective Schiff bases. In some cases, Schiff bases and their complexes have similar activity against bacteria and fungi. Chelation may enhance or suppress the biochemical potential of bioactive organic species. The higher activity of the metal complexes may be owing to the effect of metal ions on the normal cell membrane. Metal chelates bear polar and nonpolar properties together; this makes them suitable for permeation to the cells and tissues. Changing hydrophilicity and lipophilicity probably leads to bring down the solubility and permeability barriers of cell, which in turn enhances the bioavailability of chemotherapeutics on one hand and potentiality at another [27].

## Figures and Tables

**Figure 1:**
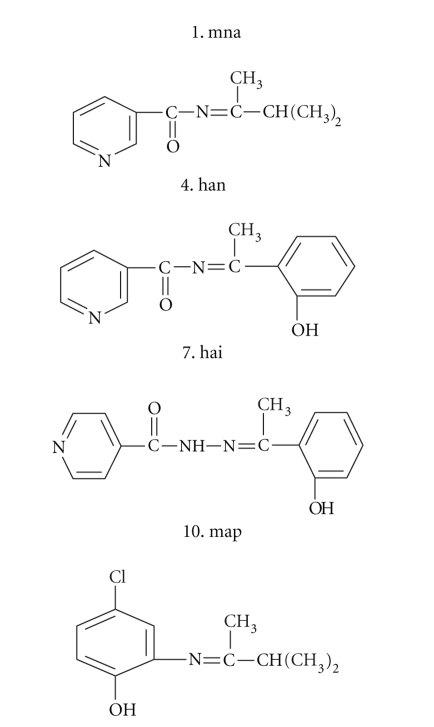
Structures of the Schiff base (Ligands).

**Figure 2:**
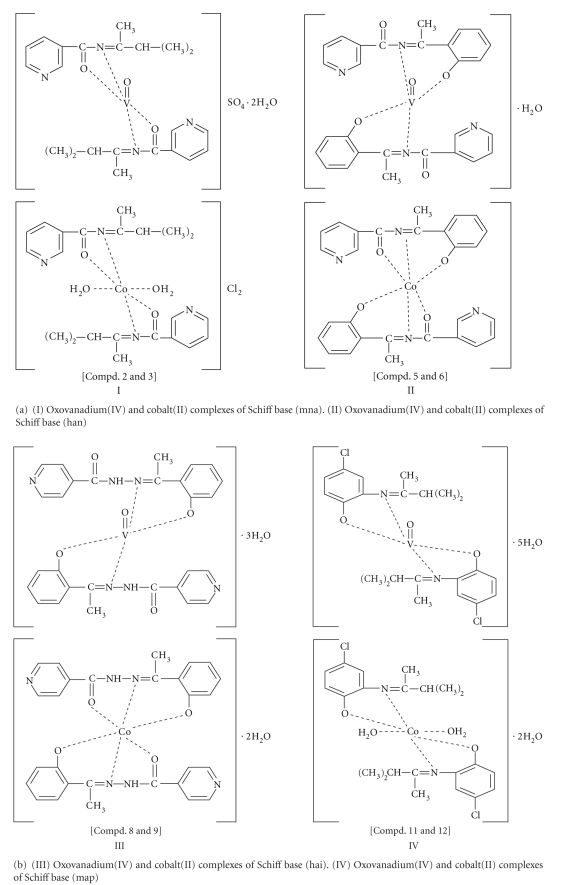
Proposed structures of the metal complexes.

**Table 1:** Analytical and physical data of ligand and metal complexes.

Compound number	Compounds/Molecular Formulae		Elemental analysis % found/(Cal.)					
	Molecular wt/colour	Dec.temp./ M.Pt. (°C)	C %	H %	N %	Yield %	*μ*_eff_ B.M	Cond. S cm^2^ mol^−1^
(1)	C_11_H_14_N_2_O (mna)	120–125	69.5	7.1	14.5	62.0	—	—
	(Pinkish Cream)		(69.4)	(7.3)	(14.7)			
(2)	[VO(mna)_2_]SO_4_ · 2H_2_O	230–239	45.9	4.5	12.7	70.7	1.76	53.8
	(Dark Green)		(45.6)	(4.8)	(9.6)			
(3)	[Co(mna)_2_(H_2_O)_2_] Cl_2_	>300	48.0	5.2	12.7	54.5	5.07	124.5
	(Purple)		(48.3)	(5.1)	(10.2)			
(4)	C_14_H_12_N_2_O_2_(han)	123	70.1	5.2	11.9	91.2	—	—
	(Cream)		(70.0)	(5.0)	(11.6)			
(5)	[VO(han)_2_] H_2_O	205–207	59.5	3.5	14.3	85.9	1.78	20.1
	(Dark Green)		(59.4)	(3.8)	(9.9)			
(6)	[Co(han)_2_]	276–278	62.4	4.1	14.3	55.3	5.12	16.9
	(Purple)		(62.3)	(4.0)	(10.3)			
(7)	C_14_H_13_N_3_O_2_(hai)	240	65.6	5.2	16.3	86.4	—	—
	(Cream)		(65.8)	(5.0)	(16.4)			
(8)	[VO(hai)_2_]3H_2_O	120–125	53.4	3.5	13.1	54.2	1.79	7.1
	(Dark Brown)		(53.2)	(3.8)	(13.3)			
(9)	[Co(hai)_2_]2H_2_O	>300	55.7	3.7	13.1	73.4	5.10	13.3
	(Light Brown)		(55.5)	(3.9)	(13.8)			
(10)	C_11_H_14_NOCl (map)	140	62.8	6.5	6.4	44.4	—	—
	(Light Coffee)		(62.5)	(6.6)	(6.6)			
(11)	[VO(map)_2_]5H_2_O	>300	45.0	4.1	5.0	88.3	1.76	6.5
	(Black)		(45.5)	(4.4)	(4.8)			
(12)	[Co(map)_2_(H_2_O)_2_] 2H_2_O	>300	47.3	4.8	5.0	60.8	5.08	18.8
	(Black)		(47.6)	(4.6)	(5.0)			

Electronic spectra.

**Table 2:** Electronic spectral data and ligand field parameters of metal complexes [8–11].

Compound number	Complexes	Transitions	Bands (cm^−1^)	Parameters 10Dq, *B*, ß, ß%, *ν*_1_/*ν*_2_, LFSE, *λ*	Geometry of the complexes
(2)	VO(II)(mna)	^2^ *B*_2_-^2^ *E* (*ν*_1_)	12722	—	Square pyramidal/trigonal bipyramidal
		^2^ *B*_2_-^2^ *B*_1_ (*ν*_2_)	19567		
		^2^ *B*_2_-^2^ *A*_1_ (*ν*_3_)	—		

(3)	Co(II) (mna)	^4^ *T*_1_ *g*(*F*)-^4^ *A*_2_ *g*(*F*) (*ν*_2_)	12484	6935, 1029, 0.91,	Octahedral
		^4^ *T*_1_ *g*(*F*)-^4^ *T*_1_ *g*(*P*) (*ν*_3_)	19607	8.12, 2.2, 66.2, −525	

(5)	VO(II) (han)	^2^ *B*_2_-^2^ *E* (*ν*_1_)	12500	—	Square pyramidal/trigonal bipyramidal
		^2^ *B*_2_-^2^ *B*_1_ (*ν*_2_)	22311		
		^2^ *B*_2_-^2^ *A*_1_ (*ν*_3_)	—		

(6)	Co(II) (han)	^4^ *T*_1_ *g*(*F*)-^4^ *A*_2_ *g*(*F*) (*ν*_2_)	12363	6868, 1003, 0.89,	Octahedral
		^4^ *T*_1_ *g*(*F*)-^4^ *T*_1_ *g*(*P*) (*ν*_3_)	19168	10.4, 2.2, 65.6, −542	

(8)	VO(II) (hai)	^2^ *B*_2_-^2^ *E* (*ν*_1_)	13000	—	Square pyramidal/Trigonal bipyramidal
		^2^ *B*_2_-^2^ *B*_1_ (*ν*_2_)	—		
		^2^ *B*_2_-^2^ *A*_1_ (*ν*_3_)	24271		

(9)	Co(II) (hai)	^4^ *T*_1_ *g*(*F*)-^4^ *A*_2_ *g*(*F*) (*ν*_2_)	16531	9183, 1004, 0.89,	Octahedral
		^4^ *T*_1_ *g*(*F*)-^4^ *T*_1_ *g*(*P*) (*ν*_3_)	20584	10.3, 2.2, 87.7, −714	

(11)	VO(II) (map)	^2^ *B*_2_-^2^ *E* (*ν*_1_)	13200	—	Square pyramidal/Trigonal bipyramidal
		^2^ *B*_2_-^2^ *B*_1_ (*ν*_2_)	—		
		^2^ *B*_2_-^2^ *A*_1_ (*ν*_3_)	24218		

(12)	Co(II) (map)	^4^ *T*_1_ *g*(*F*)-^4^ *A*_2_ *g*(*F*) (*ν*_2_)	16894	9385, 951, 0.84,	Octahedral
		^4^ *T*_1_ *g*(*F*)-^4^ *T*_1_ *g*(*P*) (*ν*_3_)	19912	15.0, 2.2, 89.7, −717	

**Table 3:** ESR parameters of the oxovanadium (IV) complexes.

Compound number	Complexes	*g*_||_	*g*_⊥_	*g*_av_	Δ*g*
(2)	[VO(mna)_2_] SO_4_ · 2H_2_O	1.9032	1.9664	1.9453	0.0632
(8)	[VO(hai)_2_] · 3H_2_O	1.9429	1.9724	1.9625	0.0295

**Table 4:** Antibacterial screening data of Schiff bases and their metal complexes. Standard = Gentamycin.

Compound number	Diameter of inhibition zone (mm) (concentrate in ppm)								
	*E.coli*			*S. aureus*			*S. fecalis*		
	25	50	100	25	50	100	25	50	100
7(S.B)	12	12	16	—	10	20	—	—	—
8(Complex)	27	31	34	11	12	13	13	13	15
10(S.B)	12	13	15	12	12	14	12	—	13
11(complex)	12	13	16	11	12	15	15	14	15
Standard	20	23	20	10	10	12	18	20	19
DMSO	—	—	—	—	—	—	—	—	—

(—) = not measurable.

**Table 5:** Antifungal screening data of Schiff bases and their metal complexes. Standard = Nystatine.

Compound number	Diameter of inhibition zone (mm) (concentrate in ppm)					
	*A. niger *			*T. polysporum*		
	25	50	100	25	50	100
7(S.B)	13	13	15	—	15	19
8(Complex)	—	—	—	11	15	17
10(S.B)	—	—	—	10	19	19
11(Complex)	—	—	—	12	13	20
Standard	—	—	—	—	—	—
DMSO	—	—	—	—	—	—

(—) = not measurable.
